# Characterizing the evolution of oculomotor and vestibulo-ocular function over time in children and adolescents after a mild traumatic brain injury

**DOI:** 10.3389/fneur.2022.904593

**Published:** 2022-07-19

**Authors:** Adrienne Crampton, Kathryn J. Schneider, Lisa Grilli, Mathilde Chevignard, Michal Katz-Leurer, Miriam H. Beauchamp, Chantel Debert, Isabelle J. Gagnon

**Affiliations:** ^1^School of Physical and Occupational Therapy, McGill University, Montreal, QC, Canada; ^2^Sport Injury Prevention Research Centre, Faculty of Kinesiology, University of Calgary, Calgary, AB, Canada; ^3^Alberta Children's Hospital Research Institute, University of Calgary, Calgary, AB, Canada; ^4^Hotchkiss Brain Institute, University of Calgary, Calgary, AB, Canada; ^5^Montreal Children's Hospital-McGill University Health Centre, Montreal, QC, Canada; ^6^Laboratoire d'Imagerie Biomédicale, LIB, CNRS, INSERM, Sorbonne Université, Paris, France; ^7^GRC 24 Handicap Moteur et Cognitif et Réadaptation, Sorbonne Université, Paris, France; ^8^Rehabilitation Department for Children With Acquired Neurological Injury and Outreach Team for Children and Adolescents With Acquired Brain Injury, Saint Maurice Hospitals, Saint Maurice, France; ^9^Physical Therapy Department, University of Tel-Aviv, Tel Aviv, Israel; ^10^Ste-Justine Hospital Research Centre, Montreal, QC, Canada; ^11^Department of Psychology, University of Montreal, Montreal, QC, Canada; ^12^Department of Clinical Neuroscience, University of Calgary, Calgary, AB, Canada

**Keywords:** mild traumatic brain injuries, pediatric, vestibulo-ocular reflex (VOR), assessment, oculomotor

## Abstract

**Background:**

Impairments to oculomotor (OM) and vestibulo-ocular reflex (VOR) function following pediatric mTBI have been demonstrated but are poorly understood. Such impairments can be associated with more negative prognosis, affecting physical and mental wellbeing, emphasizing the need to more fully understand how these evolve.

**Objectives:**

to determine i) the extent to which performance on clinical and computerized tests of OM and VOR function varies over time in children and adolescents at 21 days, 3-, and 6-months post-mTBI; ii) the proportion of children and adolescents with mTBI presenting with abnormal scores on these tests at each timepoint.

**Design:**

Prospective longitudinal design.

**Setting:**

Tertiary care pediatric hospital.

**Participants:**

36 participants with mTBI aged 6 to18.

**Procedures:**

Participants were assessed on a battery of OM and VOR tests within 21 days, at 3- and 6-months post injury.

**Outcome measures:**

Clinical measures*: Vestibular/ocular motor screening tool (VOMS)* (symptom provocation and performance); Computerized measures: *reflexive saccade test* (response latency)*, video head impulse test* (VOR gain), and *dynamic visual acuity test* (LogMAR change).

**Analysis:**

Generalized estimating equations (parameter estimates and odd ratios) estimated the effect of time. Proportions above and below normal cut-off values were determined.

**Results:**

Our sample consisted of 52.8% females [mean age 13.98 (2.4) years, assessed on average 19.07 (8–33) days post-injury]. Older children performed better on visual motion sensitivity (OR 1.43, *p* = 0.03) and female participants worse on near point of convergence (OR 0.19, *p* = 0.03). Change over time (toward recovery) was demonstrated by VOMS overall symptom provocation (OR 9.90, *p* = 0.012), vertical smooth pursuit (OR 4.04, *p* = 0.03), voluntary saccade performance (OR 6.06, *p* = 0.005) and right VOR gain (0.068, *p* = 0.013). Version performance and VOR symptom provocation showed high abnormal proportions at initial assessment.

**Discussion:**

Results indicate impairments to the VOR pathway may be present and driving symptom provocation. Vertical smooth pursuit and saccade findings underline the need to include these tasks in test batteries to comprehensively assess the integrity of OM and vestibular systems post-mTBI.

**Implications:**

Findings demonstrate 1) added value in including symptom and performance-based measures in when OM and VOR assessments; 2) the relative stability of constructs measured beyond 3 months post mTBI.

## Introduction

Children and adolescents are particularly susceptible, to mTBI/concussion (1, 2), when compared with most adult populations, with rates of pediatric mTBI estimated between 1.1 and 1.9 million each year in the USA (3), and the highest rates demonstrated among adolescents 12–17 year old (4). This may be due to anatomical, physiologic and developmental factors such as continuing maturation (5), incomplete myelination of the brain and a more flexible skeletal structure (6, 7). Environmental factors and activities of daily life in this age group can also contribute to their overall exposure to potentially high-risk situations (i.e., chaotic settings in sports/recreation participation, school yards and gym class). While most recover within 4 weeks, approximately one third of youth can suffer persistent symptoms 3 months post-mTBI (8) and 12–14% will present symptoms for more than 1 year post-mTBI (9, 10). Persisting symptoms can put children and adolescents at risk of negative physical and/or psychosocial repercussions resulting from a longer recovery process (11, 12).

While mTBI can lead to a wide range of disturbances, the impact on oculomotor (OM) and vestibulo-ocular reflex (VOR) function in pediatric mTBI has recently attracted a large amount of interest. Relevant literature has outlined high rates of reported abnormalities and/or impairments in eye movements controlled by the OM system (24–73%) (13–16) and in VOR function (43–69%) in children and adolescents (17–20). Such a high prevalence of abnormalities and/or impairments can have a significant impact on one's ability to navigate their environment and participate in recreational activities. In children and adolescents this may consequently affect their overall mental, physical, emotional and psychosocial health and wellbeing.

Abnormalities and/or impairments discussed can be quite impactful as uncompromised functioning of the OM system enables the eyes to move to allow for clear, binocular vision, as well as maintain un-impaired tracking and smooth movements of the eyes (21). The VOR and the ability to suppress this reflex allows one to maintain gaze stability on static and dynamic targets, respectively, while the head is in motion.

Impairments and/or symptoms related to OM and VOR function present in the acute phase of recovery following pediatric mTBI are predictors of prolonged recovery (18, 22). However, their evolution over time is poorly understood. Such questions have prompted recommendations for studies to measure OM and VOR function over time beyond the acute and subacute phase (23, 24) to better understand their overall recovery patterns. While a small number of studies have started to address this recommendation in pediatric mTBI populations, they almost exclusively end follow-up assessments at time of medical clearance and/or return to play (18, 19, 22, 25–27), and their focus has mostly been limited to sport-related concussion (19, 22, 25–28). In addition, these studies are heterogeneous with regards to the time from injury to initial evaluation, including samples with initial assessment in the acute time period (<10 days) (26, 27), the sub-acute time period (<4 weeks) (18, 19, 22, 25) and even portions of samples in the chronic period (>1 month) (19, 28). These limitations should be addressed to: (1) understand the evolution of OM and VOR function post mTBI during and beyond the acute/sub-acute phase in order to determine further characteristics of impairments that may persist; and (2) confirm that OM and VOR functions indeed remain uncompromised at the time of one's return to sport and daily life.

While OM and VOR studies in pediatric mTBI often include only two timepoints of interest in their results (frequently initial clinical presentation and time to recovery) and are performed through retrospective chart reviews (18, 19, 22, 25), two recent studies included data from multiple timepoints. Such studies evaluating injured individuals beyond time of initial injury and recovery are imperative as they allow recovery progress (or lack thereof) to be monitored and evaluated. Moreover, these studies provide information to ensure the recovery is complete and remains stable when the individual returns to their regular activities of daily life as this can trigger the re-emergence of certain symptoms.

The first study referenced, by Sinnott et al. (26), assessed OM and VOR-related symptom provocation initially within 10 days, at 11–21 days and followed 63 adolescent athletes with concussion to medical recovery (time to recovery of 3 groups ranged from 22.95 to 34.94 days). Assessments were conducted in the acute, subacute and prolonged/persistent phases. However, generalizability was limited (athletic populations and sport-related concussion) and the findings only speak to the evolution of these functions up to the time of medical clearance for return to activities. The second, by Zaslow et al. (28), included 3 timepoints: initial evaluation, return to play clearance and 1 month following return to play (RTP) clearance. This study produced findings demonstrating stable OM function beyond RTP clearance. However, VOR variables were limited and the sample size was small (13 adolescents) and exclusive to athletes (28).

Overall, there are very few studies examining OM and VOR function over time in youth post-mTBI (29, 30), particularly in primary school age children (6–12 years) and non-athletes (31). As the mechanism of injury influences the injury response and pathophysiological mechanisms involved (32, 33), recruiting participants with mTBI resulting from all mechanisms is of importance to allow progress to be made in understanding the heterogeneity of mTBI-response. More fully understanding the specific impairments that may compromise OM and VOR function following mTBI, the rate at which such impairments present, as well as characterizing how they resolve, will help guide treatments delivered to ultimately hasten the return to activities of daily living in these youths.

The primary objective of this study was to determine the extent to which performance on clinical and computerized tests of OM and VOR function varies over time in children and adolescents at 21 days, 3, and 6 months after a mild TBI. The secondary objective was to determine the proportion of children and adolescents with mTBI presenting with abnormal scores on OM and VOR tests at each timepoint when compared to cut-off scores pre-determined from published literature. Finally, a tertiary objective was to identify covariates that may contribute to observed changes over time.

We hypothesized that (1) performance would vary over time in tests for which higher abnormal rates of performance were recorded at initial assessment and (2) proportions with abnormal scores would decrease from initial assessment to 3 months and remain stable at 6 months demonstrating a trend toward recovery. We hypothesize that certain covariates known to influence injury response in the context of mTBI will demonstrate a significant effect on test performance.

## Methods

This study used a prospective longitudinal design to characterize the evolution of children and adolescents' performance in OM and VOR function within 21 days of injury, as well as 3 months and 6 months after a mTBI. These timepoints were selected as they represent (1) the acute-subacute period following injury when injuries are likely to recover spontaneously, (2) a time where most deficits are resolved following mTBI (3 months), and (3) a timepoint corresponding to long-term deficits (6 months). A consecutive non probabilistic convenience sample of participants were recruited from the Emergency Department and mTBI follow-up program of a tertiary care pediatric trauma center, the Montréal Children's Hospital (McGill University Health Center) as well as at the University of Calgary Sport Medicine Centre or the Acute Sport Concussion Clinic (University of Calgary). The study was approved by the Pediatric panel of the McGill University Health Center Research Ethics Board and by the Conjoint Health Research Ethics Board at the University of Calgary.

### Participants

This study was a sub-study of a larger multi-national project (the SiMPLy Rehab initiative). Participants aged 6 to 18 and assessed within 3 weeks of sustaining a physician-diagnosed mTBI (34) were included in the SimplyRehab study. Diagnosis of mTBI (as referenced) was aligned with current best practice in the Quebec trauma system. The participants included in this sub-study were those who had completed the 3 planned evaluations during the follow-up period of this larger project. Participants were excluded if any of the following were present: (i) history of a previous TBI in the preceding 6 months or any previous TBI with unresolved symptoms/impairments; (ii) presence of comorbidities that would restrict, negatively influence or prevent the participant's ability to complete the study protocol (i.e., spinal cord injury, orthopedic or neurological condition, severe visual, vestibular, or auditory deficit); (iii) medications which affect the vestibular system; (iv) participants who consented but withdrew prior to initial assessment. Prior to enrolling in the study all participants received standard acute concussion assessment/initial management from either the emergency department, a walk-in medical clinic, a pediatrician or a family physician. For the majority of participants (Montréal-based), care was guided by the Montréal Children's Hospital Concussion KiT (35), providing a plan for general activity management, return to learn and return to physical activity/sports.

### Procedures

All assessments took place at the mTBI/concussion program within the Montreal Children's Hospital (Montréal participants) or at the Concussion Lab at the University of Calgary (Calgary participants). Participants were approached, screened for inclusion and consented to participate. They then completed three evaluations over a period of 6 months. Initial assessment within 21 days, 3 month assessment within 3 and 6 month assessment within 6 months of injury. Prior to scheduled assessments at each time point, participants completed patient-reported outcome measures online. In-person evaluation consisted of clinical and computerized measures of OM and VOR function (outlined below) as well as of additional assessments of balance and gait included to better describe our sample. Sessions lasted approximately 75 min.

### Outcome measures

There is currently no gold standard measure to assess OM and VOR function post-mTBI. As such, a battery of tests was included to assess OM function, VOR response, gaze stability, as well as VOR suppression. Computerized and standard clinical assessments were included and are described below. The outcome measures included measure a wide range of unique variables. Table 1 outlines and defines the study variables, providing cut-off values for normal vs. abnormal findings in each outcome.

**Table 1:** Variables analyzed to determine changes over time in subcomponents contributing to OM and VOR function.

**Variable**	**Outcome measure**	**Components of variable**	**Abnormal cut-off (Units)**
Overall symptom provocation	VOMS	Combined symptom findings on the VOMS tasks (smooth pursuits, saccades, convergence, vestibulo-ocular reflex and visual motion sensitivity). Abnormal if participant-reported increase of ≥2 points on four combined 0–10 point symptom scales on any domain	2 or more symptoms (N/A)
Smooth pursuit performance (vertical and horizontal)	SP task from VOMS	Clinician-observed performance measured as abnormal upon presentation of catch-up saccades. Horizontal and vertical directions observed and rated separately.	Observed (N/A)
Voluntary saccade performance (vertical and horizontal)	SP task from VOMS	Clinician-observed performance. Measured as abnormal if saccade performance is observed to be/have: hypometric, hypermetric, long-latency or poor conjugacy. Horizontal and vertical directions observed and rated separately.	Observed (N/A)
Reflexive saccades	ICS Impulse software	Average latency left/right as measured by the ICS Impulse software (computerized, continuous).	>240 (ms)
Convergence performance	Convergence task from VOMS	Clinician-observed performance on near point of convergence task. Measured as abnormal if any inability of the eyes to converge synchronously.	Observed (N/A)
Near point of Convergence	Convergence task from VOMS	Distance at which participant's eyes fail to converge synchronously or at which participant sees two distinct images of the target in focus (participant's thumb). Measured distance from thumb to tip of nose.	>6 (cm)
VOR performance vertical and horizontal	VOR task from VOMS	Clinician-observed performance. Measured as abnormal if catch-up saccades were observed. Horizontal and vertical directions observed and rated separately.	Observed (N/A)
VOR suppression performance	VOR suppression task from VOMS	Clinician-observed performance on VMS task. Measured as abnormal if participant is unable to maintain gaze on thumb during body rotations.	Observed (N/A)
VOR gain (left and right)	ICS Impulse software	Defined by the vHIT test as measured by the ICS Impulse software. Right and left mean gain obtained.	<0.80
DVA (left and right)	InVision software	Defined by the DVA test as measured by the InVision Software. Right and left LogMAR obtained.	>0.3 (LogMAR)

*OM, oculomotor; VOMS, vestibular ocular motor screening tool; N/A, normal/abnormal; VOR, vestibulo-ocular reflex; DVA, dynamic visual acuity; vHIT, video head impulse test; LogMAR, logarithm of the minimum angle of resolution*.

#### Clinical outcome measures

##### Vestibular/ocular-motor screening

The VOMS (30, 36) was used as a clinical outcome measure for OM/VOR function. The VOMS is a clinical screening tool of the vestibulo-ocular and OM function that was developed specifically to assess symptom provocation (headache, dizziness, nausea, and fogginess) induced by common VOR and OM tasks in individuals who have sustained a concussion (30). The VOMS includes 7 tasks covering OM function (smooth pursuit, horizontal saccades, vertical saccades, and near point of convergence), VOR function (horizontal VOR and vertical VOR) as well as visual motion sensitivity (VMS, measuring VOR suppression). As per test protocol, prior to beginning the VOMS, each participant rated their current symptoms using a 0 (no symptom) to 10 (severe symptoms) point scale for four commonly reported symptoms: headache, nausea, dizziness, and fogginess. They then rated their symptoms following each task, and change from initial symptom score on the four symptom scales was summed to yield a total change score for each VOMS task. As previous literature has demonstrated cutoff scores of ≥ 2 total symptoms after any VOMS item to have a high rates (96%) of identifying concussion (30), and the presence of symptom provocation on at least 1 VOMS task to have negative effects on recovery (22), a variable of overall symptom provocation was used in this study. This variable was defined as an increase of 2 or more points on any task in the VOMS according to optimal change score cut-off scores recently published in Elbin et al. (37).

Clinical performance: In addition to noting the symptom provocation induced by each task, assessors also noted performance-based observations (normal-abnormal, qualitative descriptors) for each task included in the VOMS: smooth pursuit (vertical and horizontal), voluntary saccades (vertical and horizontal), convergence, VOR (vertical and horizontal) and VOR suppression (VMS task), as well as measured near point of convergence (NPC, cm). Symptom change score reported for each subcomponent was included to fulfill our secondary objective and thus described using proportions and means.

Additional clinical outcome measures to comprehensively characterize our sample at each timepoint include patient-reported outcome measures, cervical measures and balance measures (full descriptions can be found in Supplementary Table 1).

#### Computerized outcome measures

##### The saccade test of the ICS Impulse OM module

This allowed for computerized testing of reflexive saccades performed using the ICS Impulse software (39) (ICS® Impulse software, Natus®). This product's hardware is composed of accelerometers and a camera. It is combined with rapid computerized pupil tracking software. In this test the patient is required to wear goggles that project visual horizontal saccade stimuli (laser dots appearing horizontally) onto a flat surface while eye position data is captured. The variable of interest for this test was latency (38).

##### The video head impulse test using the ICS Impulse software

This test was selected to quantify VOR gain, assessing the corresponding horizontal semicircular canals. In this test, the patient is sitting, facing the wall, maintaining their gaze on a fixation dot, while the tester rotates the patient's head horizontally 10–20 degrees in short abrupt unpredictable movements left and right (39).

##### The dynamic visual acuity test using the InVision system (NeuroCom® InVision System, Natus®)

The NeuroCom InVision System (40) was selected to provide a behavioral measure of VOR. To perform this test, the patient is seated 10 feet from the screen. The software first determines the static visual acuity by having a participant respond to a series of tumbling E displays of varying sizes (stating the direction in which the E is facing: up, down, left, right) determined by an algorithm. The test is then performed dynamically (DVA test) with fixed velocity head movements at 120 deg/sec. For the duration of the DVA test, a head tracker with built in accelerometers is worn by the patient in order to precisely quantify the head velocity at which their responses occur.

### Analysis

At <21 days, 3 and 6 months (study time points), participant performance was reported as means and standard deviation for continuous variables and proportions and percentages for categorical variables (study timepoints consisted of each time of assessment and thus were categorical). To address our *primary* objective, generalized estimating equations (GEE) taking account of within-patient variation were used to estimate the effect of time on VOMS overall symptom provocation, performance on each VOMS task (smooth pursuit, horizontal and vertical saccades, convergence, horizontal and vertical VOR and VOR suppression), NPC, reflexive saccades (ICS Impulse), VOR gain (ICS Impulse) and DVA (InVision). Models included the fixed effect of time with adjustment for covariates including age, sex and time since injury at the time of initial evaluation. Odds ratios (OR) for dichotomized outcome variables and differences in continuous outcome variables were estimated with 95% confidence intervals with the significance level set at 0.05. With regards to the link functions, in the GEE logistic regression, the “logit” link function was used while in GEE linear regression the “Gaussian” link was used.

To address our secondary objective, proportions above and below previously published normal cut-off values were determined for outcome variables at each evaluation time point.

## Results

Sample descriptive characteristics are presented in Table 2. The sample included 52.8% females and 47.2% males, with a mean age of 13.98 (SD: 2.40, 7–17 years) and among which the majority were right-handed (89%). The average time from injury to initial assessment was 19.07 days (SD: 5.93, 8–33 days). Two participants slightly outside our permissible range for initial assessment (>21+7 days, participants remained in the subacute stage of recovery) remained in the analysis as their values did not differ significantly from the rest of the sample (see limitations). Injuries in our sample occurred from sport (80.6%) or recreation/other (19.4%) with the most common location of injury being the frontal (20.6%), left temporal (23.5%) and occipital region (20.6%). In our sample, 50% of participants did not have a history of previous mTBI, 25% had suffered one, and 25% had suffered two or three previous mTBI. To further characterize the sample, Table 3 contains scores for post-concussion symptoms, cervical examination, balance/vestibulospinal measures, and global outcome at each timepoint.

**Table 2:** Descriptive characteristics of sample.

**Variable**	**Mean (SD)**	***N***
	**or %**	
Age in years, sample mean (SD)	13.98 (2.40)	36
Sex, %	-	36
Female	52.8	-
Male	47.2	-
Time from injury to baseline assessment, days	19.07 (5.93)	36
Participants seen by physio >1 week prior to initial assessment	16.7	36
Any psychological disorder*, %	11.1	36
Any developmental disability**, %	13.9	36
Personal history of migraines, %	16.7	36
Participant playing a competitive sport, %	44.4	36
# of previous mTBIs, %	-	36
0	50.0	-
1	25.0	-
2	19.4	-
3	5.6	-
Mechanism of injury, %	-	36
Sport	80.6	-
Recreational play or other	19.4	-
Location of injury, %	-	34
Frontal	20.6	-
Left temporal	23.5	-
Right temporal	14.7	-
Left parietal	2.9	-
Right parietal	2.9	-
Occipital	20.6	-
Other body part or multiple locations	14.7	-

N, Number of responses obtained upon which to base relevant variable's mean or %. *Presence of anxiety, depression, sleep disorder or other. **Presence of learning disability, ADHD, dev. disorder.

**Table 3:** Post-concussion symptoms, cervical, balance, functional and global outcome measures over time (see Supplementary Table 1, for explanation of outcome measures).

**Outcome measure**	***N***	**Initial** **assessment** **Mean (SD)**	**3-month** **assessment** **Mean (SD)**	**6-month** **assessment** **Mean (SD)**
**Post-concussion symptoms**				
PCSI total (mean)				
8–12 yr/old (score out of 36)	8/8/7	7.5 (8.26)	1.63 (2.56)	4.57 (6.40)
13–18 yr/old (score out of 120)	27/25/22	20.78 (19.39)	8.12 (15.86)	8.77 (14.77)
Dizziness present on PCSI, %	34/35/35	38.2	5.7	5.6
SCAT 5 Symptoms total /132 (13–18 yr/old)	27/25/22	24.44 (22.31)	8.92 (16.86)	9.77 (16.56)
**Cervical exam**				
Neck ROM normal, %	36/36/36	97.2	100	88.9
Neck pain present, %	36/36/36	13.9	0	0
Cervical flexion rotation pain, % yes	35/36/36	11.4	0	0
Cervical flexion endurance, s	35/35/36	25.98 (14.03)	31.59 (12.83)	34.80 (14.50)
**Balance/vestibulospinal**				
Tandem best score, s	35/35/35	16.09 (6.44)	16.75 (7.38)	15.94 (5.54)
BESS score total # errors /60	35/34/35	23.74 (8.916)	19.38 (8.791)	19.71 (9.636)
FGA Score /30	34/35/35	29.47 (0.896)	29.56 (0.695)	29.63 (0.646)
**Visual/vestibular functional impact**				
DHI total score	32/34/28	21.56 (19.63)	6.53 (17.904)	4.21 (8.93)
Cardiff total score (Logits)	33/34/33	−1.65 (1.024)	−2.44 (0.805)	−2.64 (0.673)
**Global outcome**				
Glasgow outcome scale extended (pediatric)	35/33/30	2.34 (0.873)	1.21 (0.545)	1.13 (0.346)
**Peds QL total score /100**				
Child (8–12 years old)	8/8/7	80.13	90.71	94.25 (9.98)
Teen (13–18 years old)	24/26/24	75.11	91.21	93.12 (8.52)
Peds Fatigue (8–18 years old) /100	32/34/31	68.20 (21.28)	83.13 (16.03)	86.65 (13.07)
Returned to school, %	34/34/33	88.2	97.1	90.9
Pre-injury leisure activity level, yes	33/35/33	12.1	88.6	90.9
Pre-injury level of sport, yes	33/35/33	12.1	74.3	90.9

*PCSI, post-concussion symptom inventory; SCAT, sport-concussion assessment tool; DHI, dizziness handicap inventory; Peds QL, pediatric quality of life inventory; Peds Fatigue, pediatric quality of life multidisciplinary fatigue scale; ROM, range of motion; BESS, balance error scoring system; FGA, functional gait assessment*.

Table 4 presents VOMS change scores, proportions of participants with symptom provocation (≥2 point increase) and clinician-rated abnormal performance on each of the VOMS tasks, as well as mean performance on computerized outcome measures.

**Table 4:** Mean symptom change, proportions above symptom cut-offs and proportions demonstrating abnormal performance on oculomotor and vestibulo-ocular tasks.

	**Initial assessment**			**3 month assessment**			**6 month assessment**		
**Clinical measures**	**Mean symptom change (SD)**	**Proportion** >**/**= **2**	**Proportion Abnormal performance**	**Mean symptom change (SD)**	**Proportion** >**/**= **2**	**Proportion abnormal performance**	**Mean symptom change (SD)**	**Proportion** >**/**= **2**	**Proportion Abnormal performance**
VOMS overall symptom provocation	-	20.59	-	-	2.86	-	-	11.11	-
Smooth pursuit	0.09 (0.38)	3.03	-	0.17 (1.01)	2.86	-	0.08 (0.50)	2.78	-
Smooth pursuit horizontal	-	-	14.71	-	-	5.56	-	-	5.56
Smooth pursuit vertical	-	-	32.35	-	-	11.11	-	-	13.89
Horizontal saccade	0.25 (0.67)	6.25	20.59	0	0.0	8.33	0.09 (0.507)	2.86	0.00
Vertical saccade	0.22 (0.66)	6.25	41.18	0.17 (1.01)	2.86	11.11	0.09 (0.51)	2.86	11.43
Convergence	0.41 (0.95)	12.5	3.03	0.18 (1.03)	2.94	2.86	0.08 (0.50)	2.78	2.78
Horizontal VOR	0.76 (1.70)	15.15	0.00	0.26 (1.20)	2.86	5.56	0.28 (0.82)	11.11	2.78
Vertical VOR	0.67 (1.43)	12.12	0.00	0.23 (1.19)	2.86	2.78	0.08 (0.50)	2.78	0.00
VMS/VOR suppression	0.88 (1.82)	15.15	14.29	0.2 (1.02)	2.86	2.78	0.19 (0.82)	5.56	11.11
**Computerized measures**	**Mean**	**Proportions abnormal**	**Mean**	**Proportions abnormal**	**Mean**	**Proportions abnormal**
Reflexive saccade
ICS Impulse saccade latency (ms)	199.64 (32.39)	10.34	206.78 (33.42)	13.33	204.64 (25.13)	8.57
**Convergence**
Near point of convergence (cm)	4.98 (4.06)	22.22	4.26 (6.66)	22.22	4.15 (6.34)	16.00
VOR gain
ICS Impulse vHIT left	0.99 (0.127)	9.09	1.07 (0.16)	3.03	1.04 (0.13)	2.94
ICS Impulse vHIT right	0.92 (0.86)	6.06	0.94 (0.07)	3.03	0.95 (0.12)	0.00
DVA
InVision DVA LogMAR change left	0.29 (0.14)	35.48	0.27 (0.16)	32.35	0.29 (0.19)	34.29
InVision DVA LogMAR change right	0.31 (0.15)	45.16	0.26 (0.18)	29.41	0.30 (0.23)	34.29

*Additional OM/VOR variables tested range from N = 25–36. Exact N values can be found in Supplementary Table 2. VOMS: vestibular/oculomotor screening tool; VOR: vestibulo-ocular reflex; DVA: dynamic visual acuity; LogMAR: logarithm of the minimum angle of resolution. Convergence was not a computerized measure but rather the distance measured for VOMS near point of convergence task*.

For our primary objective, our GEE model adjusted change estimates and OR showed no effect of time since injury on any of the outcomes analyzed. Time since injury was therefore not considered further in the GEE models for this objective.

Outcomes showing significant change from initial assessment to 3-month assessment (indicating higher odds of demonstrating normal performance by 3-month assessment as compared to initial assessment) include VOMS overall symptom provocation (OR 9.90, *p* = 0.01), vertical smooth pursuit performance (OR 4.04, *p* = 0.03), vertical voluntary saccades performance (OR 6.06, *p* = 0.01), and VOR gain to the right (0.07, *p* = 0.01, 95% CI: 0.01–0.12). Outcomes showing significant changes from initial assessment to 6-month assessment (indicating higher odds of demonstrating normal performance by 6-month assessment as compared to initial assessment) include vertical smooth pursuit performance (OR 3.12*, p* = 0.04) and vertical voluntary saccade performance (OR 5.91, *p* = 0.01). See Table 5 for detailed results related to the GEE analysis.

**Table 5:** Change values over time odd ratio (Binary) and parameter estimates (Continuous).

**Odds ratio**	**T2 to T1 evaluation (range)**	***P*** **-value**	**T3 to T1 evaluation (range)**	***P*** **-value**
Dichotomous variables
Overall VOMS	9.90 (1.67–58.80)	0.01*	2.26 (0.69–7.41)	0.18
Horizontal SP performance	3.02 (0.50–18.12)	0.23	3.02 (0.49–18.52)	0.23
Vertical SP performance	4.04 (1.16–14.03)	0.03*	3.12 (1.05–9.26)	0.04*
Horizontal voluntary saccade performance	3.08 (0.98–9.64)	0.05	N/A	
Vertical voluntary saccade performance	6.06 (1.73–21.21)	<0.01*	5.91 (1.56–22.31)	0.01*
Convergence performance	1.11 (0.07–17.94)	0.94	1.12 (0.07–19.12)	0.94
Horizontal VOR performance	N/A	-	N/A	-
Vertical VOR performance	N/A	-	N/A	-
VOR suppression performance	6.43 (0.56–74.31)	0.14	1.35 (0.40–4.58)	0.63
**Parameter estimate of change**	**T2 to T1 evaluation (SE, 95% CI)**	***P*** **-value**	**T3 to T1 evaluation (SE, 95% CI)**	***P*** **-value**
**Continuous variables**				
Reflexive saccades	6.20 (5.52,−4.62–17.01)	0.26	4.23 (5.98,−7.50–15.96)	0.48
Convergence	−0.52 (1.05,−2.59–1.54)	0.62	−0.64 (0.99,−2.58–1.30)	0.62
vHIT left	0.02 (0.02,−0.01–0.05)	0.31	0.03 (0.02,−0.02–0.07)	0.24
vHIT right	0.07 (0.03, 0.01–0.12)	0.01*	0.05 (0.03,−0.01–0.10)	0.08
DVA left	−0.03 (0.02,−0.07–0.02)	0.27	−0.01 (0.03,−0.06–0.05)	0.78
DVA right	−0.05 (0.03,−0.10–0.00)	0.06	−0.01 (0.03,−0.07–0.06)	0.79

*T1, initial assessment; T2, 3-month assessment; T3, 6-month assessment; SP, smooth pursuit; VOR, vestibulo-ocular reflex; vHIT, video head impulse test; N/A, not applicable as there were no observed abnormalities at initial evaluation*.

*Significant p = < 0.05.

**Figure 1:**
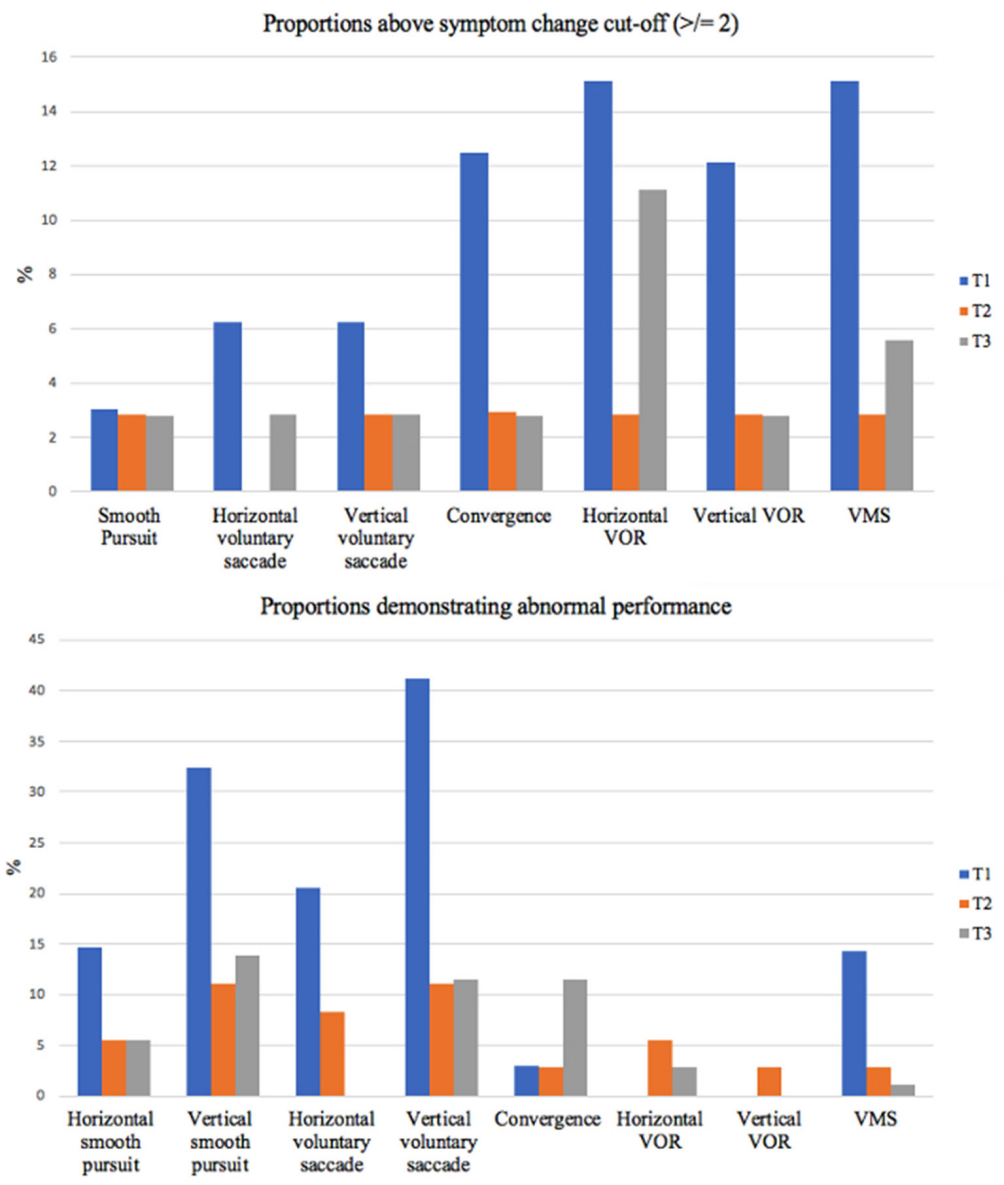
Abnormal performance and symptom provocation proportions over time. This figure outlines the proportions of our sample demonstrating symptom provocation of 2 or more (top) and abnormal performance (bottom) on each VOMS component. VOR, vestibulo-ocular reflex; VMS, visual motion sensitivity; T, timepoint.

For our secondary objective, proportions above and below cut-off values for all outcomes can be found in Table 4 and Figure 1 (VOMS symptoms only). Omitting performance on the DVA test, the saccade and smooth pursuit tasks on the VOMS had the highest proportion of observed abnormal performance, while the VOMS VOR tasks had the highest proportion of abnormal symptom provocation. Unusually high proportions of abnormal performance on the DVA InVision test were found across all three timepoints. These results will be discussed below.

For our tertiary objective, an age effect was identified on performance for the VOR suppression task on the VOMS (OR 1.43, *p* = 0.03) demonstrating that the odds of having normal VOR suppression performance increases with age. Finally, female sex increased the odds of having abnormal NPC (OR 0.19, *p* = 0.03).

## Discussion

Identifying impairments and understanding the mechanisms that contribute to persistent symptoms following pediatric mTBI can be challenging. In the past, symptom report measures have typically been used given they are simple to administer and quick and easy to interpret. However, they are not precise or objective. Moreover, in pediatric populations, concussion-related symptom reporting has been found to be inconsistent (41, 42). Specific to OM and VOR symptom provocation, 3–21% of young athletes reported symptom improvement (rather than provocation) in the VOMS while completing testing (27) reinforcing certain challenges with such measures within this age group.

This study included more targeted evaluations of OM and VOR function to elucidate whether changes or compromises to specific subcomponents of these functions may persist over time beyond symptom resolution. As such, the study included symptom-based measures and objective measures of OM eye movements and VOR responses contributing to overall OM and VOR function. Outcomes were analyzed by subcomponents in order to determine the effect of mTBI on each in this pediatric sample.

The primary objective of this study was to determine the extent to which performance on clinical and computerized tests of OM and VOR function varies over time in children and adolescents at 21 days, 3, and 6 months after a mTBI. The results demonstrated statistically significant change over time from initial assessment to follow-up assessment(s) in the specific outcomes outlined and discussed below.

The secondary objective of this study was to determine the proportion of children with mTBI presenting with abnormal scores on OM and VOR tests at each timepoint. In terms of performance, our findings demonstrated that vertical smooth pursuits and vertical voluntary saccade performance displayed the greatest abnormal proportions. In terms of symptom provocation induced, when considering both the mean symptom change and the proportions with a symptom increase ≥ 2, horizontal VOR, vertical VOR and VMS symptom provocation displayed the highest means and proportions at initial assessment. Proportions then stabilized by 3 and 6 month assessments with the percentage of abnormal proportions at these timepoints (0–11.11% across all VOMS tasks) remaining within the normative values reported in previous literature (36, 43).

The tertiary objective of this study was to identify covariates that may contribute to observed changes over time. Age and sex were found to have an effect on VMS and NPC, respectively.

### VOMS overall symptom provocation change over time

VOMS overall symptom provocation demonstrated significant change over time (*p* = 0.01). The odds of reporting symptom provocation on the VOMS assessment decreased from time of initial assessment to follow-up assessment. More specifically, the horizontal VOR, vertical VOR and VMS components of the VOMS contributed most to overall symptom provocation at initial assessment with decreasing contributions (seen through VOMS symptom change values) by 3 and 6 month assessments (Table 4). These findings are consistent with previous pediatric mTBI literature that has demonstrated a high prevalence of vestibulo-ocular dysfunctions (28.6–62.5%) (19, 44) and that has demonstrated scores on the VMS and VOR components of the VOMS to be predictive of concussion diagnosis (30).

As the VOR task targeted gaze stabilization (with active head motion) and the VMS task targeted VOR suppression through gaze pursuits while performing full-body rotations (active standing position), the symptom provocation induced may be representative of a sensory conflict (45) between the visual and vestibular systems during the tasks. The present study's results reinforce this possibility as the VOR and VMS components of the VOMS induced symptoms at initial assessment, while performance on an objective measure of VOR function (the vHIT) and clinician observed performance of VOR and VMS VOMS tasks at this same assessment were largely normal.

This sensory conflict may stem from an impairment at some point along the vestibular pathways mediating (1) the VOR response to stabilize gaze [to maintain a stable gaze it is crucial to have a fully functional VOR and intact VOR pathway (46)] and/or (2) the VOR inhibition required to generate the appropriate gaze pursuit response [in this instance to allow for gaze pursuit, neurons involved in the VOR's motor response must be attenuated or inhibited (46–48). Predictive pursuit commands contributing to VOR suppression may also play a role (49, 50)]. While outlining the specific neural pathways involved with the VOR and VOR suppression is beyond the scope of this paper, an in-depth understanding can be gained in Cullen and Roy (46), Cullen (47), Roy and Cullen (48), and Belton and McCrea (49). Future research with more precise measures could explore these findings to understand the physiological mechanisms that underly the symptoms induced. As impairments to the VOR seem to be quite responsive to targeted vestibular rehabilitation (51), understanding the mechanisms at play will allow these targeted approaches to be further refined.

### Right VOR change over time

A significant change over time was observed in right VOR gain (*p* = 0.01) as measured by the vHIT. While not significant, a similar trend was also identified in right DVA LogMAR change values (*p* = 0.06). The sample size and type of outcome measures used do not allow us to draw conclusions as to why this may be. Moreover, as previous literature found no differences in vHIT gain findings in their sample when comparing an mTBI group to a control group (52), further research is warranted to investigate the meaning as well as reproducibility (or not) of findings in the present study.

Nevertheless, previous studies outlining hemispheric asymmetries and lateralized activation patterns influenced by handedness could provide potential explanations as this study's sample contained mostly right-handed individuals (89%).

The role of handedness has been linked to different activation patterns in the brain (53), with right-handed individuals demonstrating pronounced contributions from the right hemisphere when undergoing vestibular stimulation (54). This phenomenon highlights a preference to the non-dominant hemisphere when considering vestibular function (55), consistent with findings by Bronstein et al. (56) and Arshad et al. (57) who demonstrated VOR-specific modulation dependent on their subject's handedness.

While discussing the potential role of handedness in the context of this study's findings is exploratory, future work to understand the specific role of handedness in relation to the reflexive control of eye movements could bring additional insight on this topic. Investigating the control of the VOR specifically at the brainstem and cerebellar vs. the cortical and subcortical levels would be informative as these regions are responsible for the reflexive control of gaze/head/body and self-motion/voluntary movement/balance, respectively, (58).

#### Change over time in vertical saccades and smooth pursuits

Our findings show significant change over time in performance on vertical saccade and SP tasks indicating higher odds of demonstrating normal performance by 3-month assessment as compared to initial assessment and by 6-month assessment as compared to initial assessment. These findings indicate the potential value of adding performance quantifiers when using the VOMS to assess the functioning of OM and VOR subcomponents. At the present time, there are few reliable and validated clinical measures that demonstrate the ability to quantify OM and VOR performance improvements over time following mTBI. While a previous study by Anzalone et al. (22) included additional performance quantifiers to the VOMS, it did not separate abnormal symptom provocation from abnormal clinical-observation. In our study, having both symptom provocation and clinician observation allowed the significant changes over time in performance on the vertical SP task (*p* = 0.03, 3-month assessment and 0.04, 6 month assessment) and performance on the vertical voluntary saccade task (*p* = < 0.01, 3-month assessment and 0.01, 6 month assessment) in the VOMS to be detected while also allowing us to identify the VOR contributions (previously outlined) contributing to symptom provocation. Had performance quantifiers not been included, the changes in performance on version tasks would have been overlooked. Further, it is of note that the findings of abnormal performance (clinician-observed) on vertical saccades and smooth pursuits tasks of the VOMS did not seem to translate to symptom provocation induced by these same tasks. As many would intuit observed abnormalities in performance to translate to symptom provocation, this mismatch could be explored in future research.

With regards to the directionality of the findings (vertical), this is of interest as neurophysiological contributions to saccades and smooth pursuits in the horizontal vs. vertical directions are not the same. When considering saccades, directional differences were extensively explored in the work of Irving and Lillakas (59). Four important differences were outlined: (i) the pulse innervation from the excitatory burst neurons for horizontal saccades originates from the paramedian pontine reticular formation for the horizontal direction, while this innervation originates in the rostral interstitial nucleus of the medial longitudinal fasciculus for saccades in the vertical direction (60); (ii) the neural integrator differs when considering the horizontal and vertical directions; (iii) while only two extraocular muscles are responsible for moving the eyes horizontally, four extraocular muscles must work in an integrated manner to produce vertical eye movements; and (iv) greater horizontal saccade use was demonstrated by Foulsham et al. (2011) when navigating our environments, outlining that it would be plausible to infer increased horizontal saccade pathway efficiency (59).

With regards to smooth pursuits, while the pathways share similarities, the pathway for vertical SP includes the rostral nucleus reticularis tegmenti pontis (rather than the dorsolateral pontine nuclei in the horizontal direction), involves the y-group nucleus (rather than the medial vestibular nucleus) and involves the dentate nucleus (61). Moreover, in a study by Ingster-Moati et al. (62) maturational differences were demonstrated, highlighting later maturation of the brain networks associated with vertical smooth pursuits (11 years old compared to 8 years old in the horizontal direction).

Overall, such studies along with findings of this present study underline that directional differences observed when measuring OM eye movements are important to consider when interpreting results as positive findings could hold different meanings depending on directionality.

#### Significant covariates and notable observations

All models in this study included sex, age and time since injury as potential covariates. The findings demonstrate increased odds of normal VMS with increasing age in a pediatric mTBI population. This would suggest improvements in VMS as one matures through adolescence and approaches adulthood. Such findings align with literature indicating higher rates of motion sensitivity in children, with peak incidence rates at pre-adolescence (63), and then decreasing into adulthood (64).

With regards to sex, our findings indicate increased odds of abnormal NPC in female children and adolescents with mTBI, and support similar findings by Gray et al. (20). Further research could explore potential associations between the neural pathways associated with convergence and the structural and functional differences between male and female brains highlighted in the context of concussion by Solomito et al. (65).

Of note, in this study, the high abnormal proportions in the DVA variables underline a need to further investigate: (1) if impairments to associated pathways may be present and causing these distinctively unique clinical findings, thus speaking to potential pathophysiological mechanisms at play in pediatric mTBI; (2) the reliability of the InVision DVA test when used in pediatric mTBI populations as, in addition to our findings, there are varied opinions on the reliability of the DVA InVision test (66–68) and/or; (3) the cut-off values used (this study used a cut-off value of 0.3 LogMAR). Expanding on the latter, while cut-off values of 0.2 and 0.3 are commonly used in clinical DVA assessments, these may not be adequate in the cDVA test. One particular study highlights this with a sample mean cDVA LogMAR change as high as 0.23 (0.13) when tested at 150 deg/s. in healthy NCAA division 1 athletes (69). As such a population would be expected to have superior performances to most, these findings are perplexing. A more reliable approach to cut-off scores when administering the cDVA test may be that used by Goebel et al. (70) who determined a cut-off of 0.33 LogMAR based on 2 SD from the mean of healthy control values obtained in this same study.

### Limitations

Overall, a limitation of this study is the lack of control group to provide comparative values and thus a more rigorous characterization of the change over time observed in this mTBI sample. Additionally, as with all assessments relying on clinician rating, some variability in the interpretation of participants' performance could have occurred.

Individuals recruited from the concussion clinic may have represented a population experiencing more complicated recovery. As the sample consisted of ~80% sport-related mTBI the generalizability of this study would be most suitable to similar populations. Moreover, six individuals included in this study were seen by a physiotherapist prior to their initial evaluation. While their values do not demonstrate obvious differences from the sample, this could lead to overestimating the effect of time on recovery. Finally, a small protocol deviation occurred with two individuals included in this study assessed at a time slightly beyond the permissible range due to a scheduling error. Values for these individuals did not demonstrate obvious differences from the rest of the sample and as such were included in the analyses.

With regards to our data, three participants, two at initial assessment and one at 3 month assessment demonstrated symptom improvement during the VOMS assessment. These values were removed from symptom provocation calculations as there is no physiological explanation for negative symptom reports (27) thus these would be considered to be inconsistent and unreliable (41). Further, three individuals demonstrated elevated perception times thus, according to software specifications, the validity of their LogMAR values is questionable. Lastly, as this study had a modest sample size, a type I error may have occurred where a difference was identified but may have been due to chance. Further literature is needed to confirm our findings.

## Conclusion

With increasing literature focused on OM and VOR function following pediatric mTBI, it is becoming evident that impairments to OM and VOR function may often present in a portion of this population and could influence the recovery process. This is supported by the clinical profile perspective of Kontos et al. (71), which has identified the vestibular and ocular profiles as two of the five distinct clinical trajectories following sport-related concussion. While impairments found within these profiles now often guide physical therapy interventions in mTBI settings, the evolution and precise mechanisms underlying these impairments are poorly understood. It is thus challenging to speak conclusively to the pathophysiology associated with negative prognosis. Continuing to adapt clinical assessments to include more objective rather than uniquely symptom-based measures will help to address this. Ensuring such assessments beyond medical clearance in order to monitor OM and VOR function will be equally important in order to understand whether recovery of these functions is maintained.

This study identified significant changes over time in VOMS overall symptom provocation (driven by the VOR components), vertical voluntary saccade performance, vertical smooth pursuit performance and right VOR gain. The subcomponents with the largest proportions of abnormal performance-based results were vertical voluntary saccade and vertical smooth pursuit performance. The subcomponents with the largest proportions of abnormal symptom-based results were the VOR, VOR cancellation and convergence subcomponents. Furthermore, males had higher odds of having normal NPC, and older children and adolescents had higher odds of having normal VMS performance.

The findings according to subcomponents of OM and VOR function highlight certain important observations. With regards to the VOMS, findings demonstrate: (i) the potential value of further exploring the underlying mechanisms of the VOR tasks that seem to drive overall symptom provocation on the VOMS; (ii) the value of including objective performance quantifiers to the VOMS in order to capture functional abnormalities that may be overlooked if solely relying on symptom provocation; and (iii) the value of including vertical and horizontal components when assessing SPs and saccades. With regards to the asymmetrical change in VOR gain, findings encourage exploring the potential contribution of handedness on the reflexive control of eye movements.

## Future research

Research exploring specific variables within each subcomponent outlined in this study, using computerized measures to increase the granularity of findings, and with a much larger pediatric mTBI sample would bring valuable insight to help understand the mechanisms underlying impairments to OM and VOR function in this population. Precise findings in specific variables may then encourage future studies to draw links with associated brain regions and neural circuits.

## Data availability statement

The raw data supporting the conclusions of this article will be made available by the authors, without undue reservation.

## Ethics statement

The studies involving human participants were reviewed and approved by Pediatric Panel of the McGill University Health Center Research Ethics Board and the Conjoint Health Research Ethics Board at the University of Calgary. Written informed consent to participate in this study was provided by the participants' legal guardian/next of kin.

## Author contributions

AC and IG developed the study design and methodology. AC performed the recruitment, statistical analyses, supporting statistician, and data collection. KS, LG, MC, MK-L, MB, CD, and IG supported the laboratory activities. KS, LG, MC, MK-L, MB, and CD contributed insights to discussion sections and interpretations of data analyses. IG provided extensive guidance throughout manuscript development. AC, KS, LG, MC, MK-L, MB, CD, and IG contributed to conclusion and overall manuscript content. All authors contributed to the article and approved the submitted version.

## Funding

This work was funded by the Canadian Institute of Health Research under grant number EIN 150763 and the Fonds de recherche du Québec under grant number 3679 as part of an ERA-NET NEURON JTC Cofund Program.

## Conflict of interest

The authors declare that the research was conducted in the absence of any commercial or financial relationships that could be construed as a potential conflict of interest.

## Publisher's note

All claims expressed in this article are solely those of the authors and do not necessarily represent those of their affiliated organizations, or those of the publisher, the editors and the reviewers. Any product that may be evaluated in this article, or claim that may be made by its manufacturer, is not guaranteed or endorsed by the publisher.
