# Supplementary material for: Characterizing the evolution of oculomotor and vestibulo-ocular function over time in children and adolescents after a mild traumatic brain injury
Source: Front Neurol. 2022 Jul 19;13:904593. doi: 10.3389/fneur.2022.904593 (PMC9344998; doi:10.3389/fneur.2022.904593)
Supplement: Supplementary file 1 [file Data_Sheet_1.pdf]

Supplementary Material

1 Supplementary Tables

Table 1: Description of patient-reported, cervical and balance measures

Outcome measure	Definition
**PCSI total**	Total score on Post Concussion Symptom Inventory. Developmentally appropriate and bilingual versions were used according to age. Used as a marker of recovery post-concussion [61].
**SCAT 5 total**	Sport Concussion Assessment Tool 5 total score [62].
**Dizziness present on PCSI**	Patient-reported dizziness on PCSI. Prompted additional details as to what type of dizziness was present.
**DHI total score**	Total score on Dizziness Handicap Inventory [63]. Developmentally appropriate and bilingual versions were used according to age.
**Cardiff total score**	Total score on Cardiff Visual Acuity Questionnaire (measured in Logits) [64].
**Returned to school**	Patient has returned to school
**Level of leisure**	Self-reported level at which participant is currently participating in leisure activities.
**Level of sport**	Self-reported level at which participant is currently participating in sport
**Peds QL total score**	Total score on Pediatric Quality of Life Inventory. Brings together generic core scales to measure core health dimensions outlined by the world health organization in youth [65, 66].
**Peds Fatigue**	Total score on Pediatric Quality of Life Multidimensional Fatigue Scale measuring general fatigue, sleep/rest, and cognitive fatigue [67]. Developmentally appropriate versions were used according to age.
**Glasgow outcome scale extended**	Score on the pediatric version of the Glasgow Outcome Scale measuring overall functional outcome [68].
**Neck ROM normal**	Normal neck range of motion as measured by passive head rotation, side-flexion, flexion and extension.
**Neck pain present**	Presence of pain on any of the movements assessing neck ROM.
**Cervical flexion endurance**	Length of time participant can maintain cervical flexion while lying, knees bent, hands resting on their abdomen. Participant is required to move their chin in the maximally tucked position and then lift their head approximately 2.5cm.
**Normal cervical flexion rotation**	Measures C1-C2 joint. Tested with patient in the supine position. No firm resistance encountered as examiner fully flexes the cervical spine, then rotates to the right and left with the occiput resting against the examiner's abdomen. Examiner measures disparity between right and left rotation.
**Tandem best score**	Participant's best time (seconds) on the tandem gait test performed by walking heel to toe along a 3-meter long, 38mm wide line walking forward, turning around and coming back [69].
**BESS score**	Score on Balance Error Scoring System Assessment. This test is a balance assessment protocol developed specifically for assessing concussion [70].
**FGA total**	Functional Gait Assessment test and used to assess dynamic balance.

Table 2: N values for table 4 in manuscript

	T1			T2			T3		
**VOMS components tested**	**Mean symptom change**	**Proportion >/= 2**	**Proportion Abnormal performance**	**Mean symptom change**	**Proportion >/= 2**	**Proportion Abnormal performance**	**Mean symptom change**	**Proportion >/= 2**	**Proportion Abnormal performance**
**Global VOMS symptom provocation**	-	34	-	-	35	-	-	36	-
**Smooth pursuit**	33	33	-	35	35	-	36	36	-
**SP horizontal**	-	-	34	-	-	36	-	-	36
**SP vertical**	-	-	34	-	-	36	-	-	36
**Horizontal saccade**	32	32	34	35	35	36	35	35	35
**Vertical saccade**	32	32	34	35	35	36	35	35	35
**Convergence**	32	32	33	35	34	35	36	36	36
**Horizontal VOR**	33	33	35	35	35	35	36	36	35
**Vertical VOR**	33	33	35	35	35	36	36	36	36
**VMS**	33	33	35	35	35	36	36	36	36

	T1		T2		T3	
**Additional OM/VOR tested**	**Mean**	**Proportions abnormal**	**Mean**	**Proportions abnormal**	**Mean**	**Proportions abnormal**
**Reflexive saccade ICS Impulse saccade latency**	29	29	30	30	35	35
**Convergence Near point of convergence**	27	27	27	27	25	25
**VOR gain**						
**ICS Impulse vHIT right**	33	33	33	33	33	33
**ICS Impulse vHIT left**	33	33	33	33	34	34
**DVA**						
**InVision DVA LogMAR change left**	31	31	34	34	35	35
**InVision DVA LogMAR change right**	31	31	34	34	35	35

Table 1: *N values for associated Table 4 in manuscript. As certain data was missing for different outcomes, this table provide the appropriate N values for each. VOMS: vestibular/oculomotor screening tool; VOR: vestibulo-ocular reflex; DVA: dynamic visual acuity; LogMAR: logarithm of the minimum angle of resolution*
